# Asynchronous DNA Replication of Biallelically Expressed Genes in Human Peripheral Blood Lymphocytes as a Prognostic Sign of Cancer

**DOI:** 10.17691/stm2021.13.3.04

**Published:** 2021-06-28

**Authors:** V.V. Tsepenko, T.G. Shkavrova, V.N. Cherkesov, E.V. Golub, G.F. Mikhailova

**Affiliations:** Senior Researcher, Laboratory of Molecular and Genetic Pathology, Department of Clinical Morphology; A. Tsyb Medical Radiological Research Centre — Branch of the National Medical Research Radiological Centre of the Ministry of Health of the Russian Federation, 4 Korolev St., Obninsk, 249036, Russia; Senior Researcher, Laboratory of Molecular and Genetic Pathology, Department of Clinical Morphology; A. Tsyb Medical Radiological Research Centre — Branch of the National Medical Research Radiological Centre of the Ministry of Health of the Russian Federation, 4 Korolev St., Obninsk, 249036, Russia; Head of the Laboratory for Quality Control of Medical Care; A. Tsyb Medical Radiological Research Centre — Branch of the National Medical Research Radiological Centre of the Ministry of Health of the Russian Federation, 4 Korolev St., Obninsk, 249036, Russia; Leading Researcher, Laboratory of Molecular and Genetic Pathology, Department of Clinical Morphology; A. Tsyb Medical Radiological Research Centre — Branch of the National Medical Research Radiological Centre of the Ministry of Health of the Russian Federation, 4 Korolev St., Obninsk, 249036, Russia; Head of the Laboratory of Molecular and Genetic Pathology, Department of Clinical Morphology; A. Tsyb Medical Radiological Research Centre — Branch of the National Medical Research Radiological Centre of the Ministry of Health of the Russian Federation, 4 Korolev St., Obninsk, 249036, Russia

**Keywords:** asynchronous DNA replication, fluorescence *in situ* hybridization, FISH, peripheral blood lymphocytes, *AURKA*, *TP53*, diagnosis of malignant neoplasms

## Abstract

**The aim of the study** was to identify and quantify lymphocytes with asynchronous replication of the *AURKA* and *TP53* genes in cancer patients versus controls and to assess the diagnostic capabilities of this approach.

**Materials and Methods:**

The study was carried out with peripheral blood lymphocytes probed for the *AURKA* and *TP53* genes using the interphase fluorescence *in situ* hybridization (FISH) method (Vysis, USA and Kreatech, The Netherlands). The control group included 70 people: clinically healthy donors and patients with non-oncological diseases such as gastritis, pancreatitis, chronic calculous cholecystitis, bronchial asthma, peptic ulcer disease, inguinal hernia, arthrosis, myoma, hepatitis, epilepsy, chronic prostatitis, chronic tonsillitis, and rectal adenoma. The group of cancer patients included 219 people with various oncological diseases: gastric cancer (n=68), colorectal cancer (n=30), chronic lymphocytic leukemia (n=52), Hodgkin lymphoma (n=33), and polyneoplasia (n=41).

**Results:**

In the control group, the mean frequency of lymphocytes with asynchronous gene replication (AGR) was 22.0±3.4% for *AURKA* and 18.0±3.2% for *TP53*; in the group of cancer patients, that was 36.8±4.8 and 28.4±5.1%, respectively. The excessive presence of lymphocytes with the AGR in cancer patients was consistent and statistically significant (p<0.0001). For the *AURKA* gene, the AGR-based cancer detection showed a sensitivity of 98.6±0.7%, a specificity of 100%, and an accuracy of 98.3±0.8%, and for the *TP53* gene — 78.6±3.1, 98.5±0.9, and 85.9±2.6%, respectively.

**Conclusion:**

This pilot study on lymphocytes with AGR of *AURKA* and *TP53* genes in cancer patients can serve a basis for creating a new molecular cytogenetic technology for detecting malignant neoplasms in humans.

## Introduction

Early diagnosis of malignant neoplasms is one of the most important problems of clinical medicine. In the Russian Federation, in the single year of 2018, cancer was diagnosed for the first time in 624,709 people. With the diagnosis made at stages I–II, the patient can make a recovery with a probability of about 80%. However, in Russia, only about 55% of diagnoses are made at stages I or II [1]. In many cases, cancer is detected either when people seek medical assistance for their symptoms, or during massive screening examinations aimed at detecting this specific disease. In this regard, the methodology of cancer screening is of great importance. This should be highly sensitive, specific, and near 100% accurate; in clinical practice, however, very few methods meet these requirements.

By now, the screening programs for breast, cervical, and colorectal cancers have been proved most effective. Among the detection methods, low-dose spiral computed tomography is increasingly popular. It is characterized by high sensitivity that significantly increases the likelihood of detecting even small tumors, but the method has low specificity. In addition, the patient’s body is exposed to ionizing radiation, which in itself is a risk factor for developing cancer. Of the instrumental methods for early diagnosis of cancer, ultrasound imaging should also be noted: this is the most accessible and simple modality that has no contraindications. Up to date, a lot of practical experience in using ultrasound for diagnosing cancer of the abdominal cavity and the thyroid has been accumulated.

Identifying an oncological disease at the preclinical stage is rather difficult. Therefore, in recent decades, methods of molecular genetics and cytogenetics have been increasingly used to diagnose early-stage cancers. These methods make it possible to detect abnormalities in gene expression, DNA methylation, mutations, and structural aberrations of DNA, as well as specific features of the replication processes characteristic of oncological diseases. As reported, deviations in replication timing indicate that asynchronous replication of biallelically expressed genes often correlates with the development of cancer [2–6].

In an early study [7], it was shown that the level of blood lymphocytes with asynchronous gene replication (AGR) in healthy donors did not exceed 14% for either the *TP53* gene or locus 21q22, while in patients with chronic lymphocytic leukemia, this level was twice as high for both the gene and the locus. Later, similar results were obtained in patients with solid tumors. In [8], the authors showed that in patients with renal cell carcinomas, the number of lymphocytes with AGR for the *TP53* gene and locus 21q22 was 36 and 44%, respectively, while in healthy individuals, the numbers were much lower: 12 and 14%. Subsequently, such studies were carried out with other genes both in healthy controls and in cancer patients [9].

Here, for the first time, we present the data on asynchronous replication of the *AURKA* gene in peripheral blood lymphocytes from clinically healthy individuals, patients with non-oncological diseases, and patients with several types of cancer.

**The aim of the study** was to identify and quantify lymphocytes with asynchronous replication of the *AURKA* and *TP53* genes in cancer patients versus controls and to assess the diagnostic capabilities of this approach.

## Materials and Methods

### Studied groups

The study involved a control group and a group of patients diagnosed with various cancers. The control group included 40 clinically healthy individuals and 30 patients with a history of non-oncological diseases: gastritis (6), pancreatitis (2), chronic calculous cholecystitis (11), bronchial asthma (1), peptic ulcer (1), inguinal hernia (4), arthrosis (1), myoma (1), hepatitis (1), epilepsy (1), chronic prostatitis (2), chronic tonsillitis (3), and rectal adenoma (1). Some of the patients had more than one diagnosis. In total, 34 men and 36 women aged from 21 to 77 years old (average 42) were examined.

The group of patients with cancer included 219 individuals (123 men and 96 women) aged from 21 to 88 years old (average 61). The diagnoses were distributed as follows: gastric cancer (68), colorectal cancer (30), chronic lymphocytic leukemia (52), Hodgkin lymphoma (33), and polyneoplasia (41). The polyneoplasia group included patients with two or more synchronous or metachronous tumors: melanoma, cancers of the colon, sigmoid, or ileum, gastric cancer, larynx, esophagus, kidney, lung, ovaries, breast, cervix, prostate, bladder, and thyroid cancer. The cancer stages in the examined patients varied from IA to IV.

Before taking samples, all healthy donors and non-cancer patients were informed in detail about the study; the informed consent form was signed by each participant. Studies in cancer patients were carried out according to the clinical protocols approved by the Ministry of Health of the Russian Federation and received an approval from the Ethics Committee of the A. Tsyb Medical Radiological Research Centre (Russia).

### Studied genes

*The TP53 suppressor gene.* The tumor suppressor gene *TP53*, located at locus 13.1 on the p-arm of chromosome 17, plays an important role in apoptosis, DNA repair, and cell cycle regulation in the late G1 phase. Inactivation of *TP53* is a key mechanism in malignant cell transformation.

Abnormalities of the *TP53* gene are commonly found in various human cancers. The significance of *TP53* for tumor suppression has been confirmed by demonstrating a high rate of tumorigenesis in *TP53-*deficient mice and a high risk of early cancers in humans with hereditary mutations in the *TP53* gene (Li–Fraumeni syndrome). Inactivation of this gene was noted in half of all sporadic cancers. Moreover, in cancers in which the *TP53* gene remains intact, its function is often impaired. *TP53* plays a fundamental role in controlling the cell proliferation and maintaining the integrity and stability of the genome. As reported, human malignant neoplasms are associated with genetic or epigenetic mechanisms that counteract the *TP53-*dependent signaling pathway [10–12].

*The AURKA protooncogene.* The *AURKA* gene located at locus 13.2 of the q-arm of chromosome 20, encodes for the Aurora kinase family. It is a family of serine/threonine kinases essential for normal mitosis and/or meiosis in eukaryotic cells. Aurora kinases play a crucial role in the regulation of mitotic events such as the assembly of the division spindle, cytokinesis, and the centrosome/cytoskeleton function. The *AURKA* gene is expressed in the centrosome during prophase, ensuring its division and maturation. During metaphase, this gene is located in polar microtubules and ensures the assembly of the division spindle; during cytokinesis, it is located in the Fleming’s body.

The *AURKA* gene is an oncogene. In animals, an increased activity of this gene causes genetic instability and, subsequently, tumor formation [13]. In addition, the gene is located in a region, which is often amplified in human cancers, and its mutations increase the risk of developing esophageal, ovarian, lung, and breast cancer. Abnormalities in *AURKA* expression could lead to disorders of the mitotic cycle resulting in gastric, hepatocellular, and pancreatic cancers [14–16].

### Interphase fluorescence in situ hybridization (FISH) on lymphocytes of peripheral blood

Samples of venous blood (4–6 ml) were taken using a vacuum system containing Li-heparin at a concentration of 12–30 IU per 1 ml of blood. These whole blood samples were diluted (1:9) with a warm (37°C) solution of KCl (550 mg  +  110 ml) and placed in a thermostat (37°C) for 30 min. Then, the cells were fixated in a mixture of methanol: acetic acid (3:1). Aliquots of the cell suspension (20–30 μl) were applied onto recently frozen glass slides. We used commercially available DNA probes from Vysis (USA) and Kreatech (The Netherlands) for the *AURKA* and *TP53* genes. Pre- and post-hybridization washes were performed according to the manufacturer’s instructions. A Thermobritе hybridizer (StatSpin, USA) was used for denaturation and hybridization.

### Statistical data analysis

**Figure 1:**
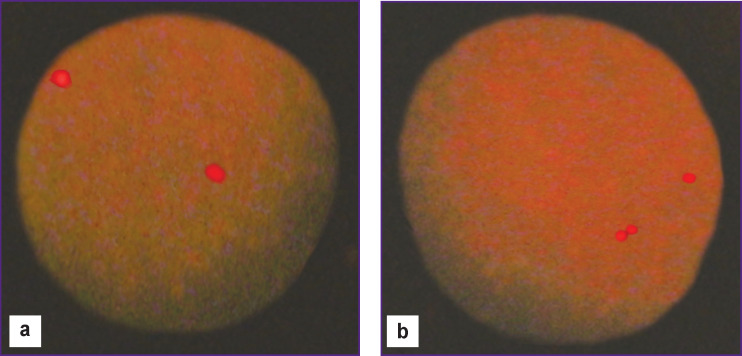
Asynchronous replication of the *TP53* gene in human peripheral blood lymphocytes: (a) normal lymphocyte (two single signals); (b) lymphocyte with impaired synchronicity of gene replication (one single and one double signal); ×1000

The slides were scored independently by two researchers using an Axio Imager A-2 fluorescence microscope (Carl Zeiss, Germany) equipped with the DAPI, Orange/Green, and Gold filters (Vysis). For each blood sample, 300–900 interphase cells with clear fluorescent signals were analyzed (Figure 1).

Statistical analysis was performed using the SPSS Statistics v. 23 and Microsoft Office Excel (2007). The resulting data were combined into variational series, for which the mean value, standard deviation, and 95% confidence interval (CI) were calculated. The data distribution was tested for normality using the one-sample Kolmogorov–Smirnov test. The sets were found homogeneous. The data were presented as mean ± standard deviation (M±σ). The significance of the differences between the mean values was assessed using one-way analysis of variance and two-tailed Student’s t-test with Newman–Keuls correction for multiple comparisons. Differences were considered statistically significant at p<0.05. To assess the informative value and the resolution power of the test, we calculated its sensitivity, specificity, and accuracy. The specificity of the method was determined from the proportion of cancer-free subjects among the “negative” analyzes; the sensitivity — as the proportion of subjects with confirmed cancer among the “positive” tests. The accuracy was calculated as the proportion of “exact matches” between the test results and the diagnoses among all subjects.

## Results

The mean frequency for lymphocytes with asynchronous replication of *AURKA* and *TP53* was higher among cancer patients than among non-cancer controls (Table 1).

**Table 1:** Occurrence of lymphocytes with asynchronous replication of *AURKA* and *TP53* in the examined groups

Groups	Number of subjects examined	Number of cells analyzed	Lymphocytes with AGR, min–max (%)	M±σ (%)
***AURKA gene***				
**Control**	**70**	**22,084**	**13.7–27.7**	**22.0±3.4**
**Cancer patients:**				
gastric cancer	65	20,083	28.6–42.0	33.9±2.9
colorectal cancer	30	9,000	32.0–44.3	35.5±2.9
polyneoplasia	41	12,404	31.3–46.0	39.6±3.8
**solid tumors (total)**	**136**	**41,487**	**28.6–46.0**	**36.3±6.8**
chronic lymphocytic leukemia	52	15,600	28.0–55.3	38.4±6.3
Hodgkin lymphoma	31	9,300	30.0–51.3	38.0±4.4
**hematology (total)**	**83**	**24,900**	**28.0–55.3**	**38.2±5.8**
***TP53 gene***				
**Control**	**65**	**20,576**	**11.7–25.3**	**18.0±3.2**
**Cancer patients:**				
gastric cancer + colorectal cancer	71	20,967	18.3–38.9	26.1±4.2
polyneoplasia	41	11,995	21.0–44.4	32.5±3.9
**solid tumors (total)**	**112**	**32,962**	**18.3–44.4**	**28.4±5.1**

For the *AURKA* gene, this value was significantly higher in all subgroups of cancer patients as compared with that in non-cancer patients or healthy subjects (p<0.05).

Among the cancer subgroups, the differences between patients with gastric cancer vs patients with polyneoplasia were statistically significant (p=0.023). There were no significant differences between the subgroups of gastric vs colorectal cancer or between chronic lymphocytic leukemia vs Hodgkin lymphoma (p=0.57 and p=0.91, respectively). After all cancer patients were combined and re-divided into subgroups of patients with either solid tumors or with oncohematology, there were still no significant differences between these two subgroups (p=0.274). Based on that, we then united these two subgroups into a single group of cancer patients.

For the *TP53* gene, the frequency of AGR in the polyneoplasia subgroup and the “gastric cancer + colorectal cancer” subgroup were significantly higher than those in control (p=0.0001 and p=0.01). There was also a statistically significant difference in the presence of lymphocytes with AGR between the patients with gastric cancer and those with multiple tumors (p=0.043).

**Figure 2:**
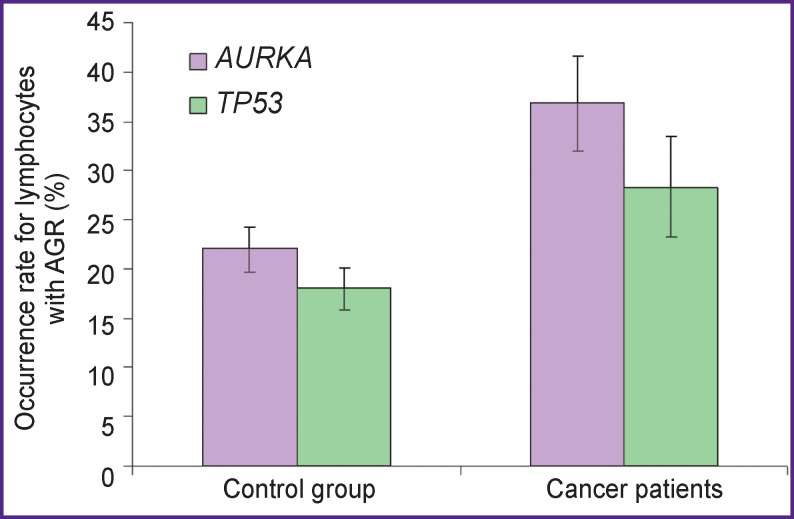
The mean frequency for lymphocytes with asynchronous replication of the *AURKA* and *TP53* genes in the control group and in cancer patients. Two-sided Student’s t-test; M±σ; p<0.05

As illustrated in Figure 2, the frequency for lymphocytes with asynchronous replication of the *AURKA* and *TP53* genes in the combined group of cancer patients (36.8±4.8 and 28.4±5.1%, respectively) significantly exceed the control levels (22.0±3.4 and 18.0±3.2%, respectively) (p=0.0001). The number of lymphocytes with AGR for *AURKA* significantly exceeded that for *TP53* both in the group of cancer patients (p=0.001) and in control (p=0.03).

As a cut-off point separating the control from the group of cancer patients, the upper limit of 95% CI of the control group was taken. For the *AURKA* gene in the control group, this value was 28.7%. In this group, there was no single person in whom the presence of lymphocytes with AGR for *AURKA* would exceed this level. In cancer patients though, the upper limit of 95% CI exceeded the 28.7% value in 214 people, and in 5 people it was below this cut-off threshold.

For the *TP53* gene, the upper limit of 95% CI in the control group was 24.2%. Only 1 person showed the value exceeding this 24.2%, and in 64 subjects of the control group, the occurrence rate of lymphocytes with AGR was within the 95% CI. Among the cancer patients, 88 individuals showed this indicator exceeding the cutoff level and in 24 patients it was below the cut-off point.

The information value and the resolution capacity of using the asynchronous replication of *AURKA* and *TP53* for making a diagnosis, are shown in Table 2.

**Table 2:** Sensitivity, specificity, and accuracy of the method (%) (M±σ)

Parameter	Gene tested	
	*AURKA*	*TP53*
Sensitivity	98.6±0.7	78.6±3.1
Specificity	100	98.5±0.9
Accuracy	98.3±0.8	85.9±2.6

## Discussion

A major characteristic of cell malignant transformation is its uncontrolled growth often caused by increased mutagenesis and genome instability [17]. Therefore, the correct order of DNA replication is essential for normal cell division to ensure that the genetic information is passed on unchanged into the next cell generation. DNA replication is a strictly regulated process; in this, most of the homologous loci in the genome replicate synchronously but a small part of them replicate asynchronously [3, 18]. Disturbances in replication timing may affect gene expression, distort epigenetic modifications, and interfere with structural rearrangements. These events can lead to genome destabilization and, ultimately, to the development of cancer [5, 19]. As shown [6], two-thirds of mutations associated with cancer result from impaired DNA replication. Analysis of genome instability, cancer and impaired replication program [2] suggests that aberrant replication is an early event in carcinogenesis.

Using the method of interphase fluorescence *in situ* hybridization, a number of research groups studied peripheral blood lymphocytes with asynchronous replication of the *TP53*, *HER-2/neu*, *C-MYC*, *RB1*, and *AML1* genes in humans and obtained the following results:

1. in clinically healthy individuals, the frequency of lymphocytes with AGR varies within 10–21% [7, 8, 20–24];
2. in cancer patients (lymphomas [24, 25], chronic lymphocytic leukemia [7], chronic myeloid leukemia [25], renal cell carcinomas [8], prostate cancer [26], and breast cancer [21]), this indicator varies within 28–41%;
3. both in oncohematological patients and in patients with solid tumors, AGR is observed in peripheral blood lymphocytes [7, 8];
4. the presence of lymphocytes with AGR increases with the grade of malignancy [23, 24];
5. the loss of synchrony of gene replication in cancer patients is a reversible epigenetic phenomenon associated, among other things, with abnormal methylation [20, 22].

Our study on lymphocytes with asynchronous replication of the *TP53* gene both in cancer-free subjects and in patients with solid tumors showed good agreement with the main findings of others [8, 24, 27]. Thus, our results on the replication timing of biallelically expressed genes in blood lymphocytes from cancer patients [9, 28], suggest that AGR can serve as a nonspecific marker of cancer.

It is known that malignant neoplasm in humans is a result of either oncogene activation or disruption of tumor suppressor genes. In this study, we selected the *AURKA* gene, which is an oncogene, and the *TP53* gene, which is a tumor suppressor gene. The results show that the frequency of lymphocytes with AGR for *AURKA* is significantly higher than that for *TP53* both in the control group and in cancer patients. Assuming that the occurrence of lymphocytes with asynchronous replication of biallelically expressed genes is a nonspecific tumor marker, we propose this indicator as a potential molecular cytogenetic test for identifying people with cancer. Table 2 shows the sensitivity, specificity, and accuracy of this test. According to the results, the most informative parameter would be the occurrence rate of lymphocytes with asynchronous replication of the *AURKA* gene since all three indices in this test exceed 98%.

## Conclusion

The level of peripheral blood lymphocytes with asynchronous replication of the *AURKA* gene can serve as a basic parameter for the development of a new molecular cytogenetic technology for detecting malignant neoplasms in humans. Creating such a technology would require further in-depth studies, in particular, the role of gender, age, smoking habits, and other factors reflecting the specific features of various cancers.
